# Characterisation of a spumavirus Gag protein

**DOI:** 10.1186/1742-4690-10-S1-P3

**Published:** 2013-09-19

**Authors:** David C Goldstone, Thomas G Flower, Neil J Ball, Marta Sanz-Ramos, Melvin W Yap, Roksana W Ogrodowicz, Nicole Stanke, Juliane Reh, Dirk Lindemann, Jonathan P Stoye

**Affiliations:** 1 Division of Molecular Structure, MRC National Institute for Medical Research, The Ridgeway, Mill Hill, London, NW7 1AA, UK; 2 Division of Virology, The Ridgeway Mill Hill, London, NW7 1AA, UK; 3 Institute of Virology, Technische Universität Dresden, Fetscherstraße 74, 01307 Dresden, UK

## Background

Human prototypic foamy virus (HPFV) belongs to the *spumaretrovrinae* subfamily and is an attractive vector candidate for gene therapy [1] as it is apathogenic. The Gag protein is not cleaved into matrix (MA), capsid (CA) and nucleocapsid (NC) as occurs in orthoretroviruses; rather, it is able to perform the roles of these proteins as a single polypeptide [2]. Foamy virus Gag proteins are targets for restriction factors such as Trim5α [3] and also interact with the aminoterminal leader peptide of the envelope protein (Env). This Gag-Env interaction is essential for budding of viral particles from the host cell [4,5].

## Materials and methods

To investigate HPFV Gag assembly and Gag-Env interactions we have undertaken combined structural and biophysical studies using X-ray crystallography, NMR, multi-angle laser light scattering (MALLS) and analytical ultracentrifugation (AUC).

## Results

An N-terminal domain (NtD) of HPFV-Gag has been identified which is functionally related to CA and MA in other retroviruses despite very low sequence homology. Structural and solution studies of the HPFVGagNtD reveal that it forms a very stable homodimer which interacts through an extended coiled-coil region. A separate region of HPFV-Gag that exhibits a propensity for selfassociation has been identified and preliminary structural and biophysical analysis suggests that it is able to form homodimers with a dissociation constant of approximately 30 µM. The molecular basis of the Gag-Env interaction has been structurally characterised and mutagenic studies, both *in vitro* and *in vivo*, have revealed the importance of the individual residues involved in this interaction.

## Conclusions

In stark contrast to orthoretroviral capsid which self-associates to form a predominantly hexameric lattice, the HPFV-Gag-NtD forms stable homodimers in solution. The identification of a secondary self-association region suggests that these dimers may undergo further multimerisation in the full-length Gag protein. Comparison of Trim5α specificity between foamy viruses suggests that the recognition site resides on the exposed surface of the N-terminal domain of the Gag protein.
